# Recent advances of cellular stimulation with triboelectric nanogenerators

**DOI:** 10.1002/EXP.20220090

**Published:** 2023-05-28

**Authors:** Xingyu Zhou, Gaocai Li, Di Wu, Huaizhen Liang, Weifeng Zhang, Lingli Zeng, Qianqian Zhu, Puxiang Lai, Zhen Wen, Cao Yang, Yue Pan

**Affiliations:** ^1^ Department of Orthopaedics, Union Hospital, Tongji Medical College Huazhong University of Science and Technology Wuhan China; ^2^ Guangdong Provincial Key Laboratory of Malignant Tumor Epigenetics and Gene Regulation, Guangdong‐Hong Kong Joint Laboratory for RNA Medicine Medical Research Center, Sun Yat‐sen Memorial Hospital, Sun Yat‐sen University Guangzhou China; ^3^ Institute of Functional Nano and Soft Materials (FUNSOM), Jiangsu Key Laboratory for Carbon‐Based Functional Materials and Devices Soochow University Suzhou China; ^4^ Department of Biomedical Engineering Hong Kong Polytechnic University Hong Kong China

**Keywords:** bioelectricity, cellular fate determination, cellular function regulation, electrical stimulation, triboelectric nanogenerators

## Abstract

Triboelectric nanogenerators (TENGs) are new energy collection devices that have the characteristics of high efficiency, low cost, miniaturization capability, and convenient manufacture. TENGs mainly utilize the triboelectric effect to obtain mechanical energy from organisms or the environment, and this mechanical energy is then converted into and output as electrical energy. Bioelectricity is a phenomenon that widely exists in various cellular processes, including cell proliferation, senescence, apoptosis, as well as adjacent cells’ communication and coordination. Therefore, based on these features, TENGs can be applied in organisms to collect energy and output electrical stimulation to act on cells, changing their activities and thereby playing a role in regulating cellular function and interfering with cellular fate, which can further develop into new methods of health care and disease intervention. In this review, we first introduce the working principle of TENGs and their working modes, and then summarize the current research status of cellular function regulation and fate determination stimulated by TENGs, and also analyze their application prospects for changing various processes of cell activity. Finally, we discuss the opportunities and challenges of TENGs in the fields of life science and biomedical engineering, and propose a variety of possibilities for their potential development direction.

## Graphical abstract

Triboelectric nanogenerators (TENGs) are portable and wearable energy collection devices. TENGs have five working modes, whose principle can be summarized as the conversion of mechanical energy to electrical energy by triboelectrification and electrostatic induction between the two materials. According to this, TENGs can be used on cell stimulation, which plays a variety of biological effects in the medical field, showing great application prospects.

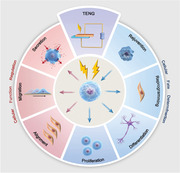

## 1 INTRODUCTION

Bioelectricity is the basic feature of biological systems and plays a vital role in the development of life science and medicine.^[^ ^1^ ^]^ These bioelectric systems regulate gene expression and participate in information transmission between cells, enabling them to determine cellular growth and form.^[^ ^2^ ^]^ The generation of bioelectricity is related to the separation formed by biological membranes and the distribution and flow of ions on both sides of these membranes. The specific proteins of cells can be activated by electrical stimulation (ES), and thus affect cellular life activities, such as cell survival, migration, proliferation, or the differentiation downstream of cells.^[^ ^3^ ^]^ Recently, ES has attracted great attention by which a variety of cellular processes can be regulated,^[^ ^4^ ^]^ such as enhancing skin repair, wound healing,^[^ ^5^, ^6^, ^7^ ^]^ inducing osteoblast and cardiac stem cell differentiation,^[^ ^8^, ^9^, ^10^ ^]^ nerve regeneration,^[^ ^11^, ^12^ ^]^ and promoting tumor cell apoptosis.^[^ ^13^ ^]^ Considering its significant effect on cell behavior, ES has made progress in accelerating wound healing, regenerating tissues, improving musculoskeletal condition, and healing bone fractures.^[^ ^5^ ^]^

At present, several devices allow the transmission of electrical signals to local cells. The concept of triboelectric nanogenerators (TENGs), a fast‐growing energy‐harvesting device that can harvest energy from environmental mechanical energy, was proposed by Wang's team in 2012.^[^ ^14^ ^]^ TENGs utilize the triboelectric effect; that is, one material will be charged after coming into contact with another material through friction. This phenomenon has been known for a long period and depends on the combination of triboelectrification and electrostatic induction between the two contact materials. TENGs have been proven to be effective in collecting environmental mechanical energy, and some research groups have used TENGs with high‐performance flexible or implantable properties to obtain mechanical energy from human motions or physiological movement in vivo.^[^ ^15^ ^]^ Owing to their advantages of high efficiency, lightweight, low cost, and easy manufacture, TENGs provide a new choice for biomechanical energy harvesting. TENGs can be developed as not only small‐scale power sources (for treatment or other medical applications), but also self‐powered sensors (for real‐time vital information monitoring) in the biomedical field.^[^ ^15^, ^16^, ^17^, ^18^ ^]^ Thus, they are widely used in the fields of self‐powered treatment of respiratory, cardiovascular, neurological, and musculoskeletal diseases; can also be used to monitor body health in real time; perform minimally invasive accurate diagnosis; enable drug delivery; etc.^[^ ^19^, ^20^, ^21^, ^22^ ^]^ TENGs are growing into the most potential biomechanical energy collector and also provide a significant opportunity for self‐powered systems construction and application in biomedical field. However, a large number of ES devices require the consideration of the problem of energy sources, and they are usually expensive and inconvenient for portable purposes.^[^ ^23^, ^24^ ^]^ TENGs are lightweight, cost effective, and easily scalable. They not only open up the field of organic nanogenerators for chemists and material scientists, but can also be applied to biomedicine and health care, providing a new paradigm in ES‐based therapeutic intervention.^[^ ^21^ ^]^

**FIGURE 1:**
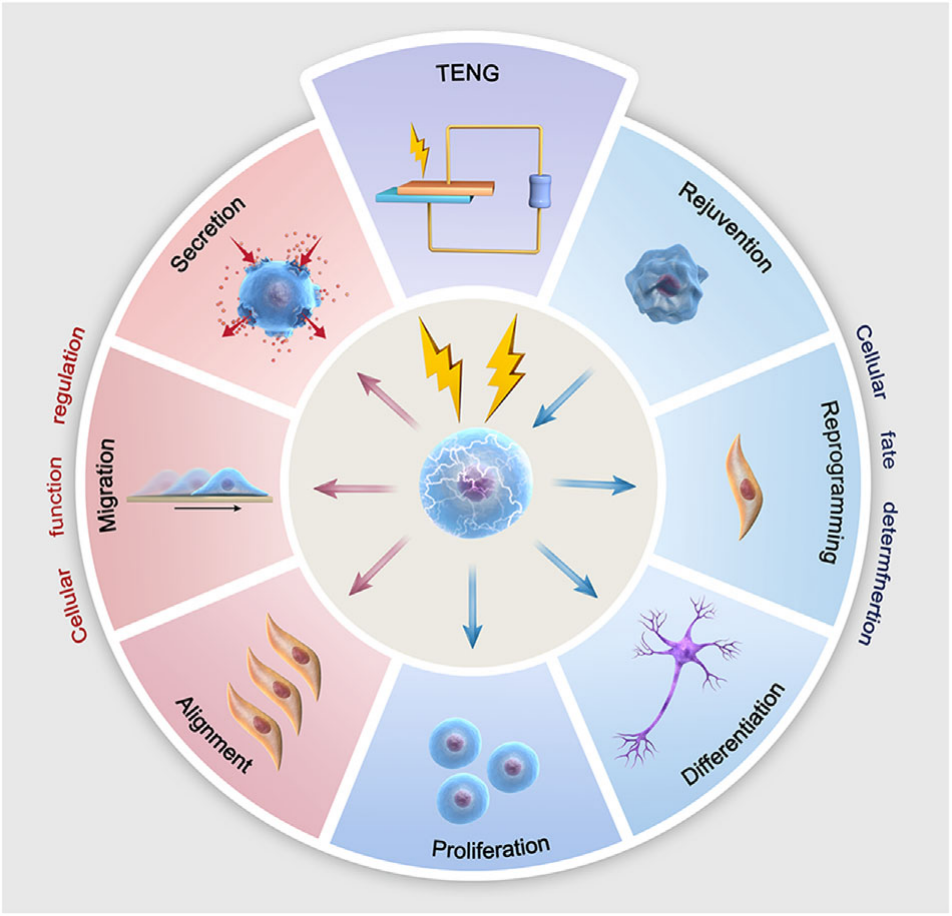
Cellular stimulation with triboelectric nanogenerators.

From this review, we will initially provide an overview of the working principle and special working modes of TENGs, and then discuss their relevant applications in vivo and in vitro. We will then summarize the function regulation and fate determination of cells stimulated by TENGs, including research progress in these fields, corresponding uses, challenges, and limitations (Figure 1). Finally, we will conclude with a perspective on the future of the development of TENGs as an emerging technology in nano‐energy field, including the opportunities and challenges of its application in neurology, regeneration therapy, rehabilitation, pharmacy technology, and others.

## 2 THE BASIC THEORY AND WORKING MODES OF TENGS

### 2.1 The basic theory of TENGs

Coupling triboelectrification and electrostatic induction is the basic principle of TENGs. Although the apparent mechanism of triboelectrification is still unclear, the triboelectrification effect which has long been known is a process of charging that occurs based on friction of two different materials when they contact each other.^[^ ^15^, ^25^ ^]^ When dissimilar materials contact each other and an external force acting causes friction, because of the different electron gain and loss abilities of the different materials, that is, their abilities to capture electrons at the material interface are different, the charge will be transferred from one material to another, resulting in the accumulation of charge.^[^ ^15^, ^19^, ^26^ ^]^ The principle of TENG current generation is based on Maxwell's displacement current theory. In short, the time‐varying electric field will lead to a change in the electric flux and then produce a displacement current.^[^ ^25^ ^]^ Due to the action of electrostatic induction, electrodes of the two materials separated from each other generate a potential difference that drives electrons to flow between the positive and negative electrodes. In this way, TENGs convert environmental mechanical energy, which provides contact and separation changes, into electrical energy.^[^ ^15^, ^19^, ^26^ ^]^ However, the ES generated by TENGs in response to dynamic mechanical stimuli is in the form of a pulse, which is different from the constant current stimulation generated by AC or DC output.^[^ ^18^ ^]^

### 2.2 The working modes of TENGs

According to the “contact” mode of the two friction materials and the linkage of different external circuits, there are four working modes for TENGs, including mode of vertical contact separation (CS), lateral sliding (LS), single electrode (SE), and freestanding triboelectric layer (FT),^[^ ^19^, ^27^ ^]^ which have their own unique advantages, characteristics, and applications. For these four basic modes, under periodic mechanical triggering, TENGs can obtain a continuous AC output based on their working principle.^[^ ^19^, ^27^ ^]^

Recently, Wang's team invented a TENG with constant current called DC‐TENG, which can obtain a constant current output and directly supply power to electronic devices.^[^ ^28^, ^29^ ^]^

#### 2.2.1 Vertical contact‐separation mode

**FIGURE 2:**
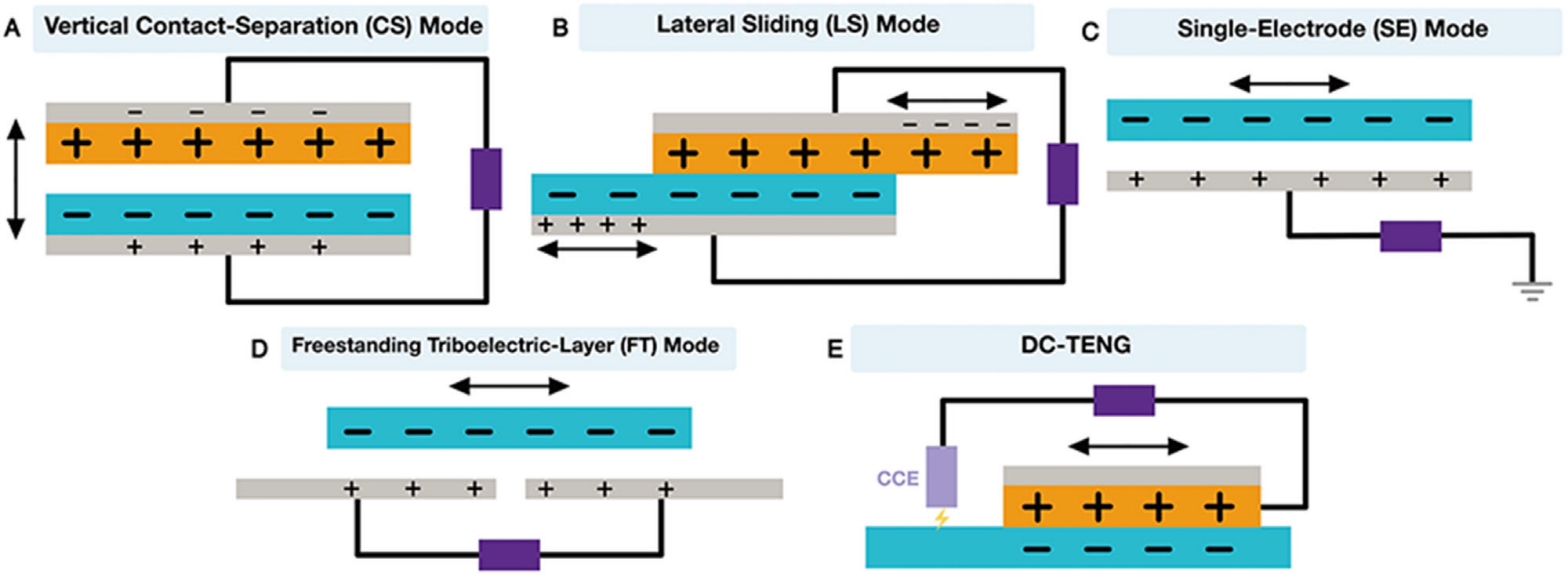
Five working modes of triboelectric nanogenerators (TENGs). (A) Vertical contact‐separation (CS) mode. (B) Lateral sliding (LS) mode. (C) Single‐electrode (SE) mode. (D) Freestanding triboelectric‐layer (FT) mode. (E) DC triboelectric nanogenerator (DC‐TENG).

The CS mode is shown in Figure 2A as an example. There are two electrodes stacked face to face. They have different dielectric properties and are deposited on the outer surfaces of two friction layers which create opposite charges after contact.^[^ ^19^, ^25^ ^]^ When the external force separates two electrodes, the capacitance between the two electrodes with air as the medium is formed. With the increasing space between the electrodes, the electric potential between the two contact surfaces changes. When a load circuit connects the two electrodes, electrons can form a current through the circuit. In contrast, the electric potential difference formed by the induced charge will disappear as the two dielectric films make contact again, and the electrons will return through the closure of the air gap between the two electrodes. The contact and separation of electrodes periodically and vertically will produce AC output.^[^ ^19^, ^28^ ^]^ As the most basic mode, it has advantages such as a simple structure, convenient manufacture, strong robustness, and high instantaneous power density.^[^ ^15^, ^19^, ^25^, ^27^, ^28^ ^]^

#### 2.2.2 Lateral sliding mode

The initial structure of the LS mode is similar to that of the CS mode, except that the LS mode is induced by relative sliding between friction layers (Figure 2B). When the two friction surfaces contact, their relatively parallel sliding results in a friction charge generated on the two surfaces. With the periodic process of sliding separation and closure, the LS mode of TENGs produces AC output.^[^ ^19^, ^25^, ^30^ ^]^ Compared with the CS mode, this mode is conducive to packaging because there is no air gap between the two electrodes. Compared with simple contact, friction charge generation is more efficient, which can significantly increase the output performance.^[^ ^15^, ^27^, ^31^ ^]^ However, the LS mode also has disadvantages; that is, the wear of TENGs caused by high‐frequency friction is severe, which will reduce the durability and robustness.^[^ ^15^, ^19^, ^25^, ^27^, ^28^ ^]^

#### 2.2.3 Single‐electrode mode

Both CS and LS modes have a disadvantage. They require two electrodes connected through external circuits, which may limit the universality and applicability of TENGs in obtaining energy, especially when collecting energy from frequently and freely moving objects.^[^ ^32^ ^]^ The invention of the SE mode breaks this limitation. It only requires a grounded electrode to generate AC output induced by the periodic movement of charged objects (Figure 2C).^[^ ^19^, ^25^ ^]^ This model has a simple structure that is easy to manufacture, which greatly broadens the range of TENGs applications. However, the output performance of this mode is low, because the electrostatic shielding effect due to the electrode limits the electron transfer, resulting in a reduction in efficiency.^[^ ^15^, ^19^, ^25^, ^27^, ^28^ ^]^

#### 2.2.4 Freestanding triboelectric‐layer mode

The FT mode consists of two symmetrical electrodes under a dielectric film (Figure 2D), which is the optimization of the SE mode.^[^ ^32^ ^]^ An external load circuit is used to connect the two electrodes, and a small gap is left between them and the friction layer of the dielectric film. The dielectric film reciprocating motion causes a change in the electric potential difference between the two electrodes, while the electrons generate current back and forth between the two electrodes.^[^ ^25^, ^33^ ^]^ Since there is no direct friction or contact between the dielectric layer and the electrode, this mode can significantly reduce the wear of the friction layer, greatly improve the durability of TENGs, and prolong their service life. Moreover, the efficiency of power conversion can theoretically achieve 100%, which also benefits from the absence of friction.^[^ ^15^, ^19^, ^25^, ^27^, ^28^ ^]^

#### 2.2.5 DC triboelectric nanogenerator

The above four modes of TENGs cannot provide power to electronic equipment or energy storage components directly without rectification by a rectifier circuit because of the AC output they produce. Lately, a derived TENG producing constant DC current (DC‐TENG) was developed, which produces DC output through triboelectrification coupling and electrostatic breakdown (Figure 2E).^[^ ^29^ ^]^ It also adopts a horizontal sliding pattern to generate friction charges. The difference is that researchers creatively added a charge collection electrode (CCE) and used the artificial lightning generated by electrostatic breakdown to transport electrons from the triboelectric layer (PTFE) to the CCE. In this way, CCE is connected with another friction electrode (FE) through an external circuit to generate periodic DC under the periodic sliding of the sliding block where the FE is located.^[^ ^28^ ^]^ The DC‐TENG converts mechanical power into a DC current, which can directly supply power to electronic equipment or charge an energy storage unit without a rectifier. Therefore, this system has broad application prospects, and there will be further research conducted to study the detailed corresponding mechanism and precise theoretical model.^[^ ^28^, ^29^ ^]^

## 3 ACTIVITY CHANGES OF CELLS ELECTRICALLY STIMULATED BY TENGs

Bioelectricity is a widespread phenomenon in nature. Regarding cells, it can control cellular behavior and morphogenesis, and ES can also manipulate cellular responses.^[^ ^34^, ^35^ ^]^ Bioelectricity means the slowly changing ion flow generated and perceived by all types of cells, which mediates the communication between cells and the communication between cells and the intracellular environment through the ion channels, ion pumps, and gap junctions between adjacent cells, allowing ion flow‐mediated signals to propagate and diffuse in cell populations.^[^ ^36^ ^]^ All cells, not just neurons, have ion channels and pumps that are used to control their resting potential. On this basis, the transcription of downstream genes can be regulated by voltage changes. Bioelectrical signals are transformed into the intracellular signals of second‐messenger cascades to play regulatory roles, such as proliferation, differentiation, apoptosis, and migration, in various cells.^[^ ^2^, ^37^ ^]^ The ES produced by TENGs can affect a variety of cellular processes, including cellular function regulation (alignment, migration, secretion, membrane permeability change, etc.) and cell fate determination (proliferation, differentiation, reprogramming, rejuvenation, etc.). Therefore, cracking the bioelectric code of cells and using the effects of electrical signals to transmit information in the development of biophysical tools, such as TENGs, for regulating cell activities will have a revolutionary impact on the fields of biology, medicine, and bioengineering.^[^ ^2^, ^36^, ^38^, ^39^, ^40^ ^]^

### 3.1 Cellular function regulation

Electric and electromagnetic fields are able to have influences on various membrane functions, such as ligand binding, ion channels, density, and distribution changes of receptors, resulting in cell activity regulation.^[^ ^41^ ^]^ In the following, we will illustrate how the ES of TENGs is used to regulate cell function in four aspects: cell alignment, migration, secretion, and changes in membrane permeability.

**FIGURE 3:**
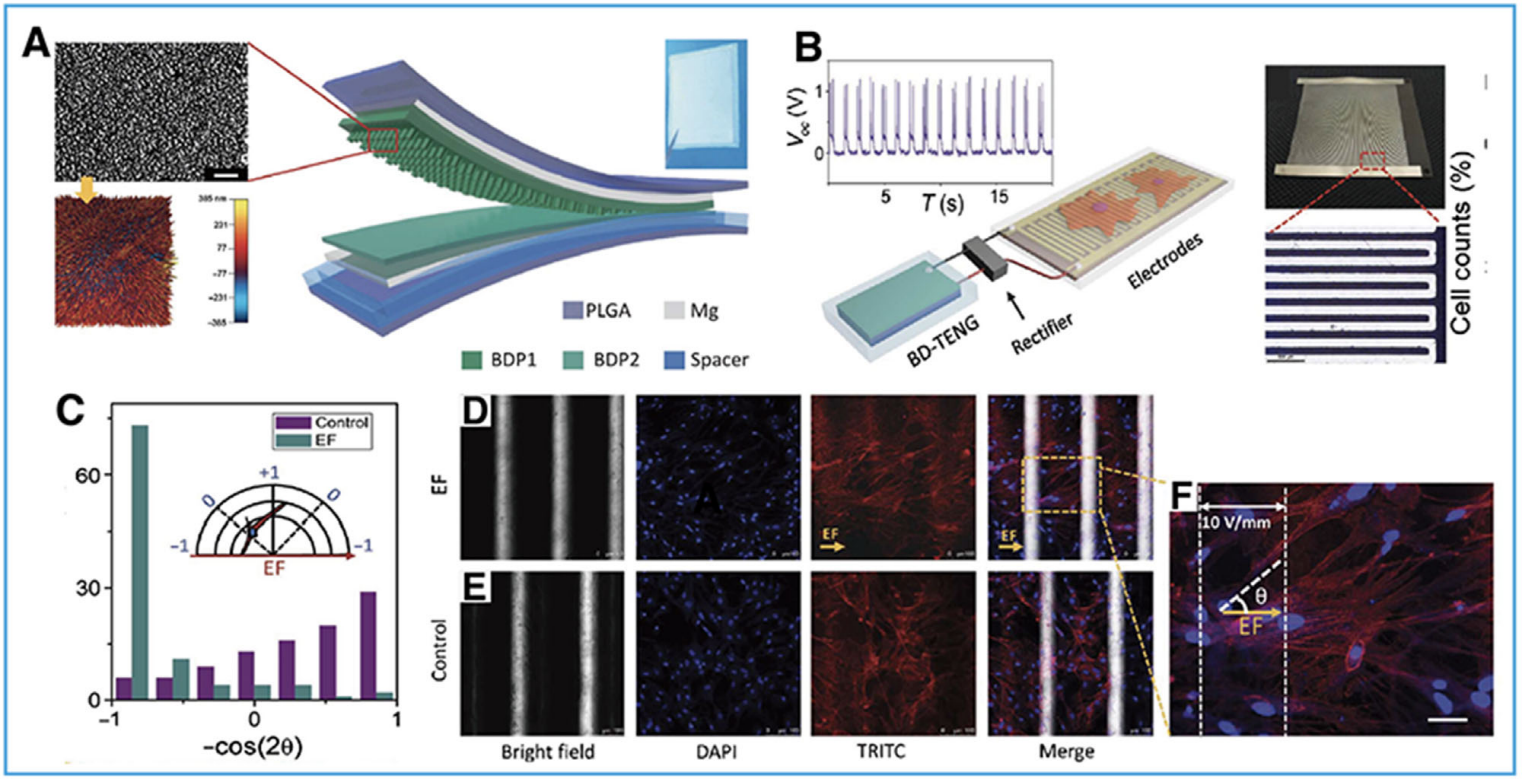
Effects of ES generated by triboelectric nanogenerators (TENGs) on cell alignment. (A) Structural design of BD‐TENGs, with images of the appearance and nanostructure of the BDP film. (B) Schematic illustration of the self‐powered nerve cell stimulation system. (C) Cell alignment analysis. (D–F) Nerve cells appearance cultured on the electrodes. Reproduced with permission.^[^ ^43^ ^]^ Copyright 2016, American Association for the Advancement of Science.

Cell alignment is the basis of the effective differentiation and maturation of cells. Studies have shown that cell structure and alignment directional tension may affect protein channel activation and gene expression, and thereby affect the normal growth and repair process of tissues and organs.^[^ ^42^ ^]^ TENGs have been proven to be of immediate practicability with regard to inducing the directional alignment of neuronal cells. Zheng et al.^[^ ^43^ ^]^ used biodegradable materials to produce TENGs of the CS mode (Figure 3A) and combined them with an ES device for electric field‐assisted neuron cell orientation. Primary neurons were stimulated repeatedly by the ES device after 24 h of culture, applying electric field treatments of 10 V mm^−1^ at 1 Hz for 30 min day^−1^. The observation results of laser scanning confocal microscopy showed the orientation trend of the alignment to be parallel to the electric field of neurons, and most of the neurons stimulated by the electricity had good orientation (Figure 3B–E). Thus, the ES of TENGs significantly guides the alignment of neurons, which has an important influence on nerve repair.^[^ ^43^ ^]^

**FIGURE 4:**
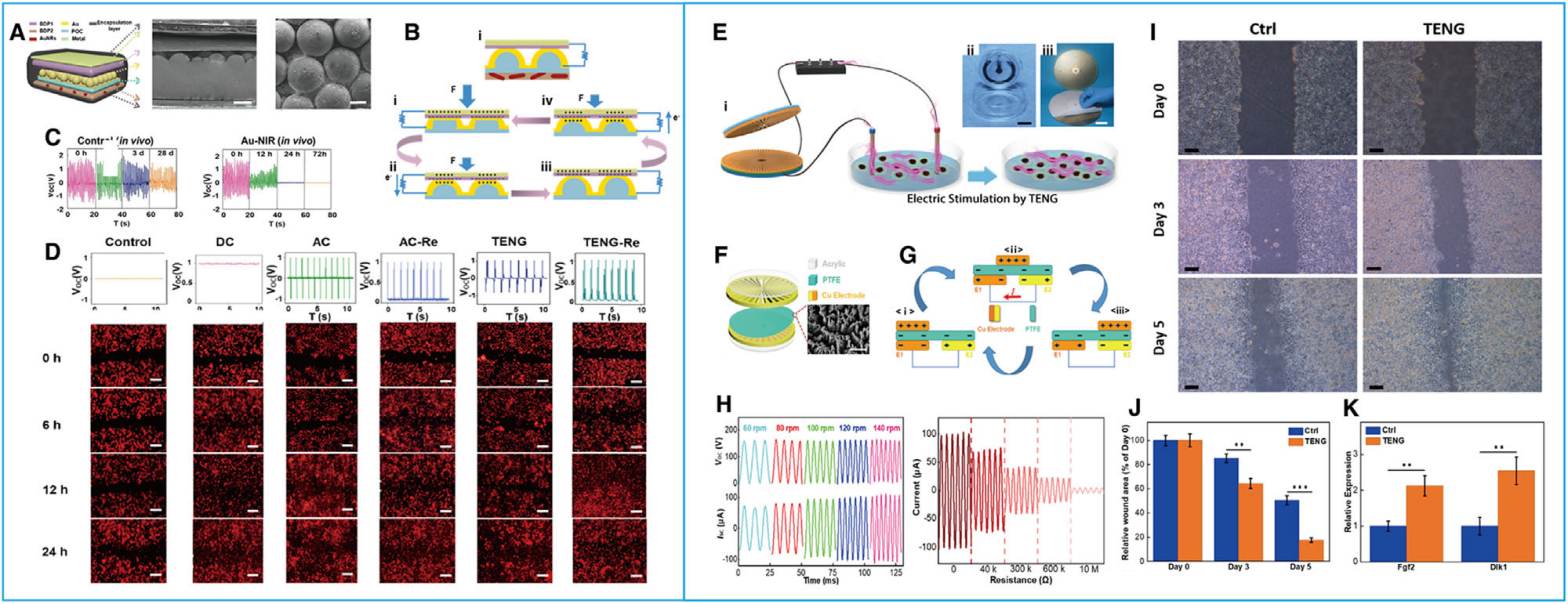
Effects of ES generated by triboelectric nanogenerators (TENGs) on cell migration. (A) Structural design of BD‐iTENGs with a section view and top view. (B) Working principle of the BD‐iTENGs. (C) In vivo degradation evaluation of BD‐iTENGs. (D) Fluorescence microscopy images of cell migration after ES. Reproduced with permission.^[^ ^46^ ^]^ Copyright 2018, Elsevier. (E) Schematic illustration of the ES system. (F) Structural design of RD‐TENGs. (G)Working principle of the RD‐TENGs. (H) The output performance of RD‐TENGs. (I) Scratches in L929 cells stimulated by RD‐TENGs. (J) Quantitative analysis of the migration results. (K) mRNA expression of Fgf2 and Dlk1 in RD‐TENG‐stimulated cells. Reproduced with permission.^[^ ^47^ ^]^ Copyright 2018, Elsevier.

Directed cell migration usually depends on a variety of external signals. The galvanotaxis of cells enables cells to respond to electric field signals from the surrounding environment and migrate along the potential gradient. The whole migration process involves the joint action of the extracellular matrix and cytoskeleton. For example, endogenous electric fields generated at epithelial wounds will lead to the migration of endothelial and neuronal cells for the repair of damaged tissue.^[^ ^44^, ^45^ ^]^ Li et al.^[^ ^46^ ^]^ created a tunable biodegradable implantable TENG (BD‐iTENG) that was capable of responding to near‐infrared (NIR) photothermal manipulation (Figure 4A,B) made from gold nanorods (AuNRs), which can respond to NIR light to accelerate degradation (Figure 4C). Without NIR light treatment, the iTENGs were used to stimulate fibroblast cells (L929), and the cells of the treatment group obviously migrated to the middle of the scratch (Figure 4D).^[^ ^46^ ^]^ In 2019, Hu and his team^[^ ^47^ ^]^ designed a rotatory disc‐shaped TENG (RD‐TENG), which was mainly composed of two parts fitted coaxially in operation, a disc‐shaped rotator and the stator. The rotator was a print circuit board (PCB) which was disc shaped with radial Cu deposition, while the stator was another PCB‐coated interdigital Cu electrode adhered to a PTFE film (Figure 4E–G). The device provided a variable AC signal over a wide range of voltage and current (Figure 4H). They used RD‐TENGs at a 50 μA current to act on L929 cells and found that two genes related to migration, both fibroblast growth factor 2 (Fgf2) and delta like noncanonical notch ligand 1 (Dlk1), were significantly upregulated in the stimulated cells, and increased 67% of the cell migration rate compared to that of the control sample (Figure 4I–K). These results indicate that RD‐TENGs can promote cell migration, which also provides an application basis of TENGs for portable wound healing systems.^[^ ^47^ ^]^

**FIGURE 5:**
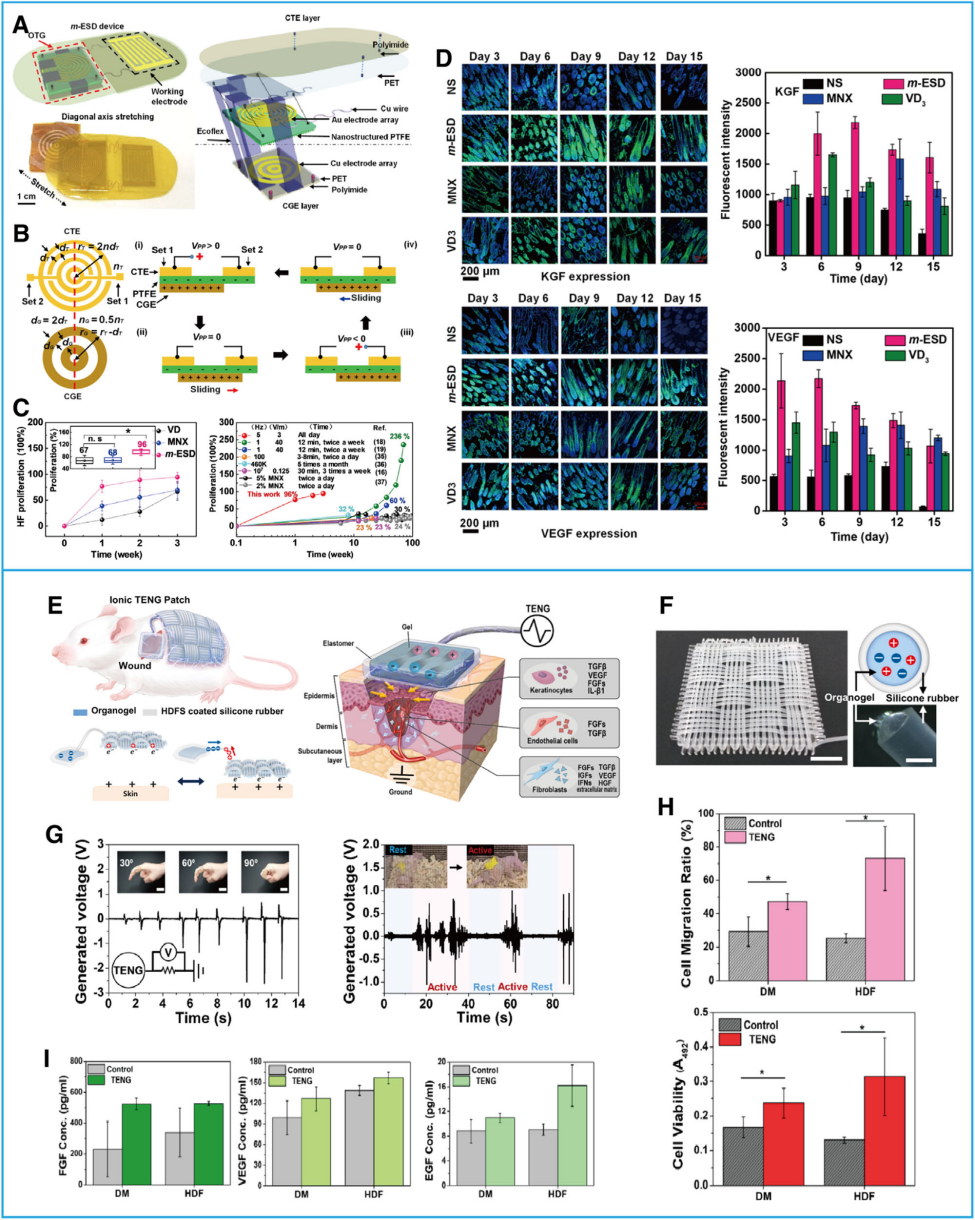
Effects of ES generated by triboelectric nanogenerators (TENGs) on cell secretion. (A) Structural design of a motion‐activated electric stimulation device (m‐ESD). (B) Geometric parameters and working principle of the m‐ESD. (C) Evaluation of HF proliferation after treatment. (D) Expression levels of keratinocyte growth factor and vascular endothelial growth factor (VEGF) (confocal imaging and quantitative analysis). Reproduced with permission.^[^ ^49^ ^]^ Copyright 2019, American Chemical Society. (E) Schematic illustration and working principle of an ionic TENG (iTENG) patch. (F) Photograph of the ionic fabric. (G) Output performances of iTENGs. (H) Evaluation of cell migration and cell viability after treatment with the ES of iTENGs. (I) Expression levels of FGF, VEGF, and EGF after treatment with the ES of iTENGs. Reproduced with permission.^[^ ^50^ ^]^ Copyright 2020, Elsevier.

The influence of the electric field on ion channels and gap junctions indicates that intercellular communication may be regulated by biophysical stimulation, and the change in membrane potential has a certain impact on cell secretion.^[^ ^48^ ^]^ Some tissue cells, such as muscle, ligament, bone, and cartilage, have been proven to be able to respond to biophysical stimulation including electric and electromagnetic fields, and increase the expression of extracellular matrix proteins, including a series of complex and continuous processes such as protein transcription, assembly, transportation, and secretion.^[^ ^41^ ^]^ Yao et al.^[^ ^49^ ^]^ made a motion‐activated and wearable ES device (m‐ESD) (Figure 5A,B) based on a noninvasive biological effect named electrotrichogenesis (ETG) induced by ES, which can enhance calcium influx into dermal papilla cells through voltage‐gated transmembrane ion channels and stimulate protein synthesis and cell division. They found that when treated with the m‐ESD, SD rats had higher hair follicle density and longer hair shaft length compared to rats treated with traditional drugs such as vitamin D3 and minoxidil; thus, the device promoted hair proliferation (Figure 5C). In addition, for genetically defective nude mice, the device improved the secretion of vascular endothelial growth factor (VEGF) and keratinocyte growth factor (KGF), alleviated the disorder of hair keratin, reconstructed the regeneration microenvironment, and promoted hair regeneration (Figure 5D).^[^ ^49^ ^]^ Recently, Jeong et al.^[^ ^50^ ^]^ designed a wearable ionic triboelectric nanogenerator (iTENG) patch (Figure 5E). This was a textile‐type iTENG composed of an organogel and elastomeric microtubular structure. To achieve ionic conductivity, they added lithium chloride (LiCl) into the organogel, which was then woven with microtubules to form a fabric entity (Figure 5F). Electric current was produced between the ionic fabric and the dermal surface through the contact and separation, which made the iTENGs output alternating current to skin tissue (Figure 5G). Experiments showed that in addition to enhancing the migration and proliferation of fibroblasts (Figure 5H), ES induced by iTENGs also increased the expression and secretion of biological molecules including TGF, VEGF, FGF, and EGF (Figure 5I). Therefore, this device has great potential in the treatment of severe chronic wounds.^[^ ^50^ ^]^

**FIGURE 6:**
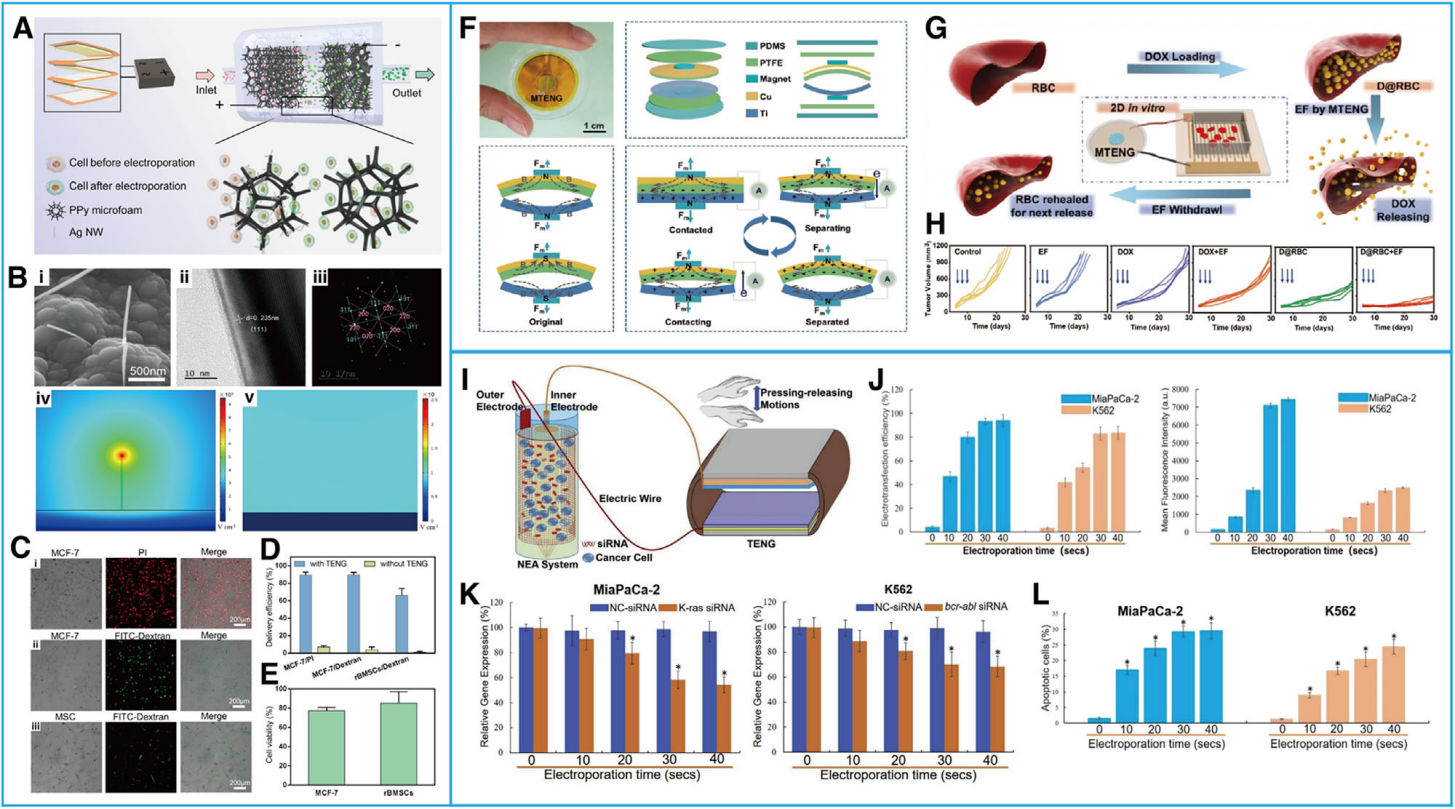
Effects of ES generated by triboelectric nanogenerators (TENGs) on changing cell membrane permeability. (A) Schematic illustration of a TENG‐driven high‐throughput electroporation system. (B) Images and electrical field distribution of the Ag NW‐modified PPy microfoam. (C) Biomolecular delivery of various types of cells (D) Quantitative delivery efficiency analysis of MCF‐7 cells and rBMSCs. (E) Cell viability analysis of MCF‐7 cells and rBMSCs. Reproduced with permission.^[^ ^21^ ^]^ Copyright 2020, American Chemical Society. (F) Structural design and working principle of the MTENGs. (G) Schematic illustration of the process by which MTENGs work. (H) Evaluation of the antitumor ability of MTENG‐controlled DDS treatment (in vivo tumor growth curve). Reproduced with permission.^[^ ^55^ ^]^ Copyright 2019, WILEY‐VCH. (I) Schematic illustration of a T‐NEA device. (J) Evaluation of siRNA delivery efficiency after treatment with T‐NEA. (K) Evaluation of gene transcription and expression levels in different cells. (L) Evaluation of the relative bioactivity of cells after electrotransfection with siRNA. Reproduced with permission.^[^ ^56^ ^]^ Copyright 2019, Elsevier.

The cell membrane is the main protective barrier that isolates the cell from the internal environment. However, when a certain external electric field is applied to the cell, the rearrangement of lipid molecules on the cell membrane results in nanoscale electropores, which is a process called electroporation, so that the membrane permeability is increased and water‐soluble molecules and ions can be exchanged through the cell membrane.^[^ ^51^ ^]^ The pores sizes are related to the application time of the electric field, the line tension on the pore perimeter, the surface tension of the cell membrane, and the induced transmembrane potential.^[^ ^52^ ^]^ This also provides a feasible method for clinical targeted drug delivery into cells.^[^ ^53^ ^]^ However, traditional electroporation technology has some limitations because of the large equipment required, its low efficiency and the resulting great damage to cells.^[^ ^54^ ^]^ Liu et al.^[^ ^21^ ^]^ designed a multilayered TENG with high‐throughput and self‐powered electroporation (Figure 6A), which is based on the “lightning‐rod effect.” They fabricated a polypyrrole (PPy) microfoam electrode, modifying it with silver nanowires, which could increase the local electric field strength and reduce the accompanying cellular damage (Figure 6B). When the external electric field of 20 V, 5 Hz, and 200 pulses acted on cells through the electrode, the cellular membrane permeability increased, and exogenous bioactive substances could enter the cytoplasm (Figure 6C–E) to meet the demand of TENG‐driven electric field‐based drug delivery.^[^ ^21^ ^]^ In 2019, Zhao and his colleagues^[^ ^55^ ^]^ produced a magnet triboelectric nanogenerator (MTENG) to induce a change in the membrane permeability of doxorubicin (DOX)‐loaded red blood cells (RBCs) when reaching tumor tissue to increase DOX release and achieve the purpose of killing cancer cells (Figure 6F–H). Yang et al.^[^ ^56^ ^]^ designed a new TENGs‐driven nanowire electrode array (T‐NEA) platform, which can deliver siRNA to cells and play a tumor‐suppressing role by silencing specific oncogenes (Figure 6I–L). All of these devices have application prospects for clinical cancer treatment.

### 3.2 Cellular fate determination

The signal produced by exogenous electrical activity can regulate the gene expression of cells to drive cells to complete various activities in the stage of growth and development.^[^ ^57^ ^]^ Next, we will review the cases of TENGs used to intervene in cell proliferation, differentiation, reprogramming, and rejuvenation.

**FIGURE 7:**
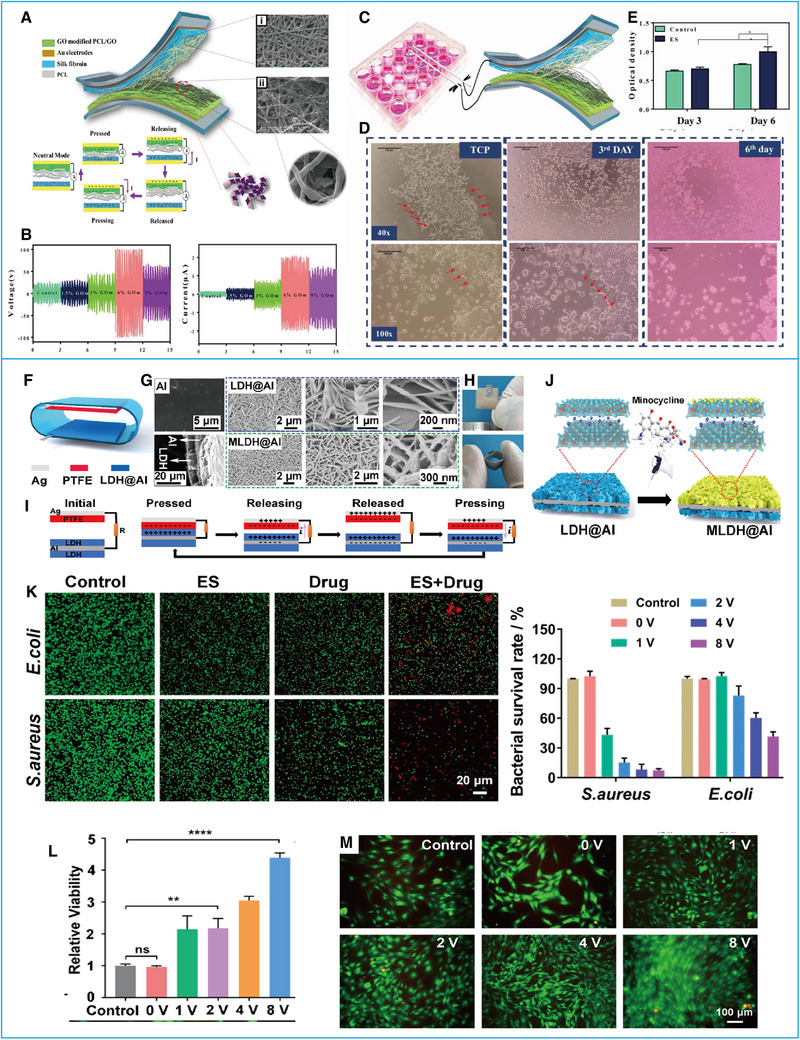
Effects of ES generated by triboelectric nanogenerators (TENGs) on cell proliferation. (A) Structural design and working principle of GOm‐SF‐based TENGs. (B) Output performances of GOm‐SF‐based TENGs. (C) Schematic illustration of the ES acting on neural cells in vitro. (D) Optical electron microscopy images of PC12 cells. (E) Cell viability evaluation of the PC12 cells. Reproduced with permission.^[^ ^58^ ^]^ Copyright 2020, IOP. (F) Structural design of the surface‐engineered TENGs (SETENGs). (G) SEM images of Al foil, LDH@Al film, and LDH@Al film with minocycline (MLDH@Al). (H) Photographs of SETENGs. (I) Working principle of SETENGs. (J) Schematic illustration of drug loading on the electrode of an SETENG. (K) Evaluation of antibacterial behaviors after the action of SETENGs. (L) Cell proliferation stimulated by different intensities of ES. (M) Fluorescence microscopy images of fibroblasts in different states stimulated by different intensities of ES. Reproduced with permission.^[^ ^59^ ^]^ Copyright 2021, Elsevier.

Cell proliferation is fundamental to cellular activity and function. The signals generated by electrical activities can be transmitted through ion channels and signal transduction receptors and regulate cell proliferation.^[^ ^57^ ^]^ In 2020, Parandeh and his co‐workers^[^ ^58^ ^]^ developed a flexible TENG that consists of a silk fibroin (SF) fibrous layer and a polycaprolactone (PCL)/graphene oxide (GO) fibrous layer (Figure 7A). The authors adopted a PCL/GO layer with various amounts of GO nanosheets for surface modification, which markedly improved the TENGs output. These TENGs were able to produce 100 V voltage, 3.15 mA m^−2^ current, and a 72 mW m^−2^ power density (Figure 7B). After continuous stimulation for 20 min day^−1^ with an alternating 0.5 V cm^−1^ output voltage and 1.4 μA output current, PC12 cells showed significant proliferation and migration (Figure 7C–E), showing the potential of these TENGs as a power supply for biomedical applications.^[^ ^58^ ^]^ Du et al.^[^ ^59^ ^]^ proposed a TENG patch whose surface of electrode was modified into Mg‐Al layered double hydroxides (LDH) used to load drug. The two friction layers compose an arch patch with an electronegative PTFE and electropositive Mg‐Al LDH@Al film as the electrode, which can load and release minocycline during treatment (Figure 7F–J). Therefore, this surface‐engineered TENG patch can play an antibacterial role (Figure 7K), and studies have confirmed that the ES produced by TENGs can promote the proliferation of fibroblasts (NIH‐3T3 cells) in wounds (Figure 7L,M). Thus, this application provides a possible solution for the treatment of infected wounds.^[^ ^59^ ^]^

**FIGURE 8:**
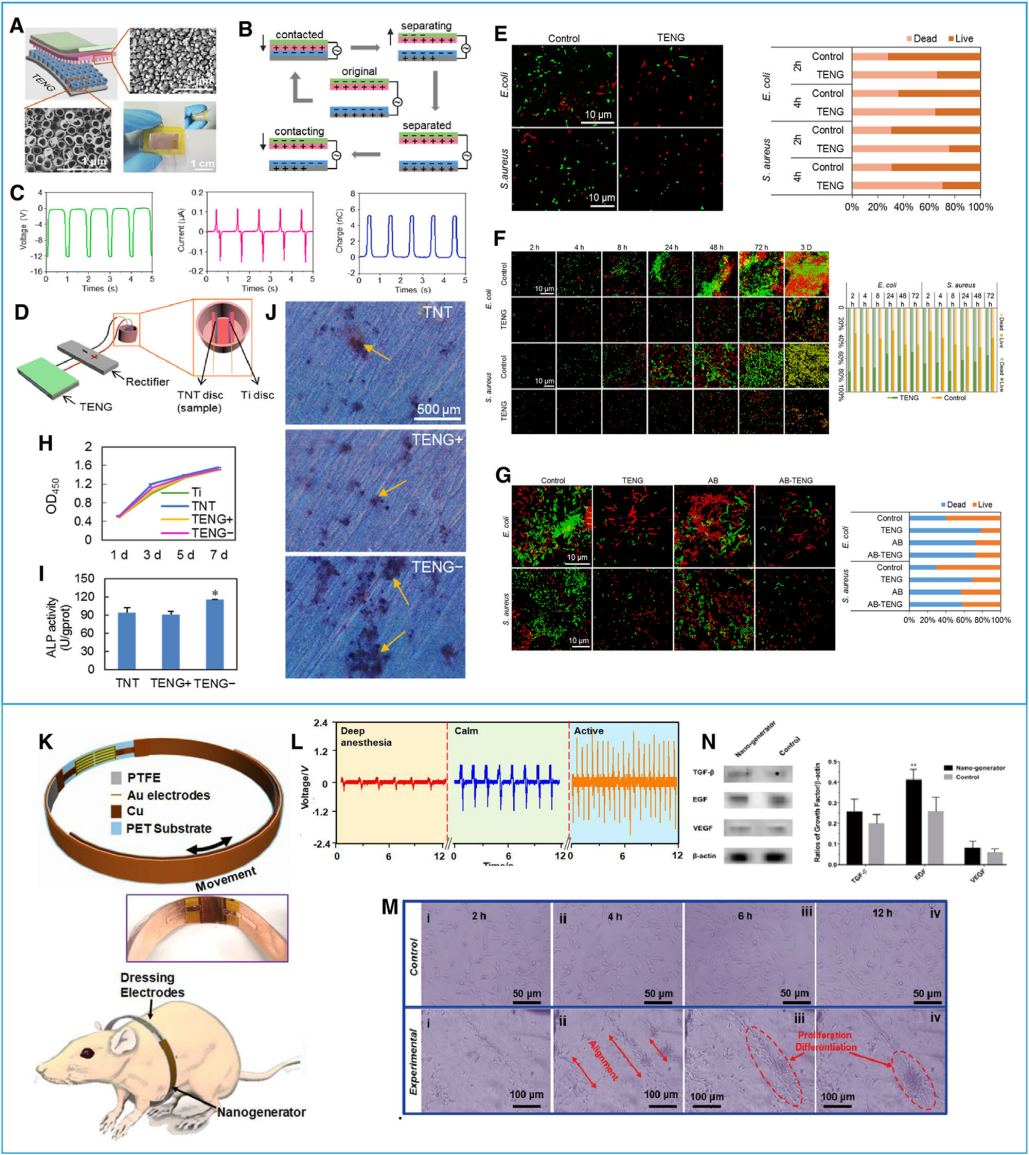
Effects of ES generated by triboelectric nanogenerators (TENGs) on cell differentiation. (A) Structural design of TENGs with an image of friction layers. (B) Working principle of TENGs. (C) Output performances of TENGs. (D) Schematic illustration of experimental devices. (E) Evaluation of initial bacterial adhesion after ES (SYTO 9/PI staining image and the ratio result of live and dead bacteria). (F) Evaluation of antibiofilm formation effects after ES treatment (LIVE/DEAD staining image and the ratio result of live and dead bacteria). (G) Evaluation of the elimination of formed bacterial biofilm after ES (LIVE/DEAD staining image and the ratio result of live and dead bacteria). (H) Evaluation of the viability and proliferation of preosteoblast MC3T3‐E1 cells by CCK‐8 assay. (I) ALP activity test of MC3T3‐E1 cells after treatment for 7 days. (J) Calcium deposits of MC3T3‐E1 cells after treatment for 21 days. Reproduced with permission.^[^ ^62^ ^]^ Copyright 2020, Elsevier. (K) Structural design of TENGs and schematic illustration of biomechanical energy harvesting from the mechanical action of rat respiration. (L) Output performances of TENGs. (M) Cell morphology analysis of proliferation and differentiation. (N) Expression levels of growth factors. Reproduced with permission.^[^ ^5^ ^]^ Copyright 2018, American Chemical Society.

The process of cell differentiation in organisms is strictly limited by gene regulation. For example, when the body is damaged, stem cells are guided to develop in a specific direction to repair damaged tissues and maintain the integrity of the morphology and function of organisms.^[^ ^60^ ^]^ There is a close relationship between the metabolic state and substances in the body and the expression of differentiation genes. The changes in metabolic substances in internal environment trigger a series of phosphorylation cascades, activating downstream regulatory molecules to regulate cell differentiation.^[^ ^61^ ^]^ Based on CS mode, Shi et al.^[^ ^62^ ^]^ manufactured a TENG, which operates through the repeated contact and separation between the PTFE layer and TiO_2_ and generates an induced current with a 12 V open‐circuit voltage, 0.15 μA short‐circuit current, and 5.3 nC transferred charge (Figure 8A–D). These TENGs have certain antibacterial properties and osteogenesis‐promoting effects. Anodized titanium with a negatively charged implant surface can significantly inhibit the growth of gram‐positive and gram‐negative organisms and inhibit the formation of biofilms (Figure 8E–G). When TENGs were used to stimulate preosteoblastic cells (MC3T3‐E1) on the negatively charged surface, the calcium deposition of bone cells increased markedly compared to that of others, indicating that except for the obvious adhesion and proliferation, the negatively charged surface is also more conducive for MC3T3‐E1 cells to differentiate into osteoblasts (Figure 8H–J).^[^ ^62^ ^]^ In 2018, Long et al.^[^ ^5^ ^]^ developed wearable TENGs that can obtain energy from mechanical respiratory movement (Figure 8K,L), which can accelerate wound healing in rats. It consists of an LS‐mode TENG which converts biomechanical energy and its load electrodes. These TENGs are made of an electronegative Cu/PTFE layer and another electropositive Cu layer stacked on different sides of a polyethylene terephthalate (PET) substrate, respectively. They can generate a 2 V cm^−1^ electric field at a frequency of 1 Hz, which enhances the alignment, migration, and proliferation of fibroblasts and promotes the expression of transforming growth factor beta (TGF‐β), epidermal growth factor (EGF), and VEGF. They also promoted the differentiation of fibroblasts into myofibroblasts, providing a contraction force for wound closure (Figure 8 M,N).^[^ ^5^ ^]^ Another study^[^ ^9^ ^]^ pointed out that ES can activate cellular calcium signaling pathways and interact with PKC/ERK pathway networks to mediate the expression of related transcription factors and structural genes, promoting the cardiomyocyte differentiation of human induced pluripotent stem cells (hiPSCs) and improving differentiation efficiency. Therefore, ES‐preconditioned hiPSCs have broad application prospects and research value in the treatment of myocardial infarction. However, the specific mechanism and whether the TENGs can provide ES for treatment need to be further studied.

**FIGURE 9:**
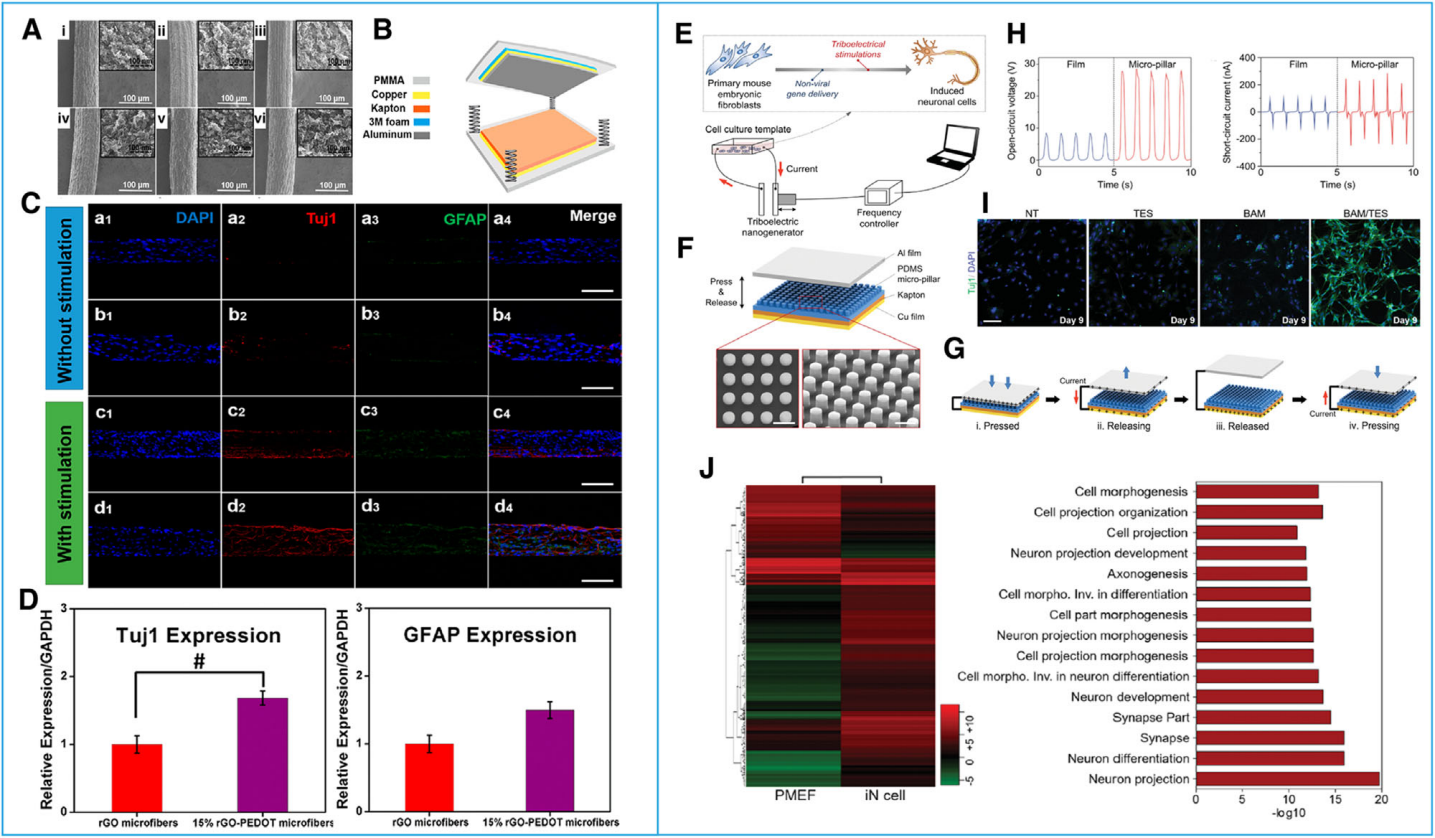
Effects of ES generated by triboelectric nanogenerators (TENGs) on cell reprogramming. (A) SEM images of microfibers made from GO suspension PEDOT solution at different concentrations. (B) Schematic illustration of the TENGs self‐powered ES‐assisted neural differentiation system. (C) Merged fluorescence image comparison between immunostained cells with and without ES on two types of microfibers. (D) qPCR analysis of the cell expression levels of Tuj1 and GFAP on the two types of microfibers. Reproduced with permission.^[^ ^66^ ^]^ Copyright 2016, American Chemical Society. (E) Schematic illustration of the experimental setup of TES for cell reprogramming. (F) Structural design with SEM images of TES. (G) Working principle of the cycle of the electricity generation of TES. (H) Output performances of TES including open‐circuit voltage and short‐circuit current at a 1 Hz pulse frequency. (I) Expression of Tuj1 in cells of each group after ES acting. (J) Analysis of global transcriptional profiles after ES. Reproduced with permission.^[^ ^67^ ^]^ Copyright 2016, Wiley‐VCH.

In the body, maintaining the cellular identity of terminally differentiated cells is very important for the maintenance of normal tissue function, but these cells still retain a certain ability of dedifferentiation and transdifferentiation, which is called cell plasticity. Dedifferentiation refers to the transformation of differentiated cells into cells with greater developmental potential, while transdifferentiation refers to the transformation of cells into another mature cells.^[^ ^63^ ^]^ Under certain created experimental conditions or when the body responds to injury, controllable plasticity changes, that is, reprogramming, may occur through the activation of specific signaling pathways or as a response to substances released during injury or inflammation. This process has great therapeutic potential.^[^ ^64^, ^65^ ^]^ In 2016, Guo and his co‐workers^[^ ^66^ ^]^ designed a self‐powered ES‐assisted system to regulate the mesenchymal stem cells (MSCs) neural differentiation. The system consisted of two parts: TENGs that provided high‐efficiency pulsed ES signals and graphene oxide (RGO) hybrid microfibers reduced by poly(3,4‐ethylenedioxythiophene) (PEDOT) as scaffolds (Figure 9A,B). The results showed that neural special markers for neuron cells (Tuj1) and for glial cells (GFAP) in MSCs cultured on the 15% rGO–PEDOT hybrid microfiber expressed more than on the rGO microfiber, indicating that the enhancement in the MSCs neural differentiation was stronger than that for glial differentiation by adding PEDOT (Figure 9C,D). The 15% rGO–PEDOT hybrid microfiber induces MSCs to preferentially differentiate into neuronal phenotypes.^[^ ^66^ ^]^ Also in this year, Jin et al.^[^ ^67^ ^]^ established a biphasic triboelectric stimulation platform for cell reprogramming. A triboelectric stimulator (TES) was connected to conductive titanium (Ti)‐deposited silicon (Si), which has high conductivity (Figure 9E–G). These TESs could provide two‐phase pulse electrical signals with an open‐circuit voltage (*V*_oc_) of approximately 30 V as well as a short‐circuit current (*I*_sc_) of approximately 270 nA (Figure 9H) to realize the nonviral transformation of primary mouse embryonic fibroblasts (PMEFs) into induced neuronal (iN) cells. Under the stimulation of the TESs, the genes encoding the neuronal lineage‐specific TFs Brn2, Ascl1, and Myt1 L (BAM TFs) were transferred to the PMEFs through the transport of engineered nanoparticles and electroporation. Then, the transdifferentiation of the stimulated PMEFs was significantly accelerated. The results revealed that the combined application of TESs and nanoparticles effectively promoted the PMEFs direct conversion to produce functional neuron cells with a mature phenotype (Figure 9I,J). This study showed that TENGs have great application prospects in the cell replacement therapy of diseases, especially neurodegenerative diseases.^[^ ^67^ ^]^

**FIGURE 10:**
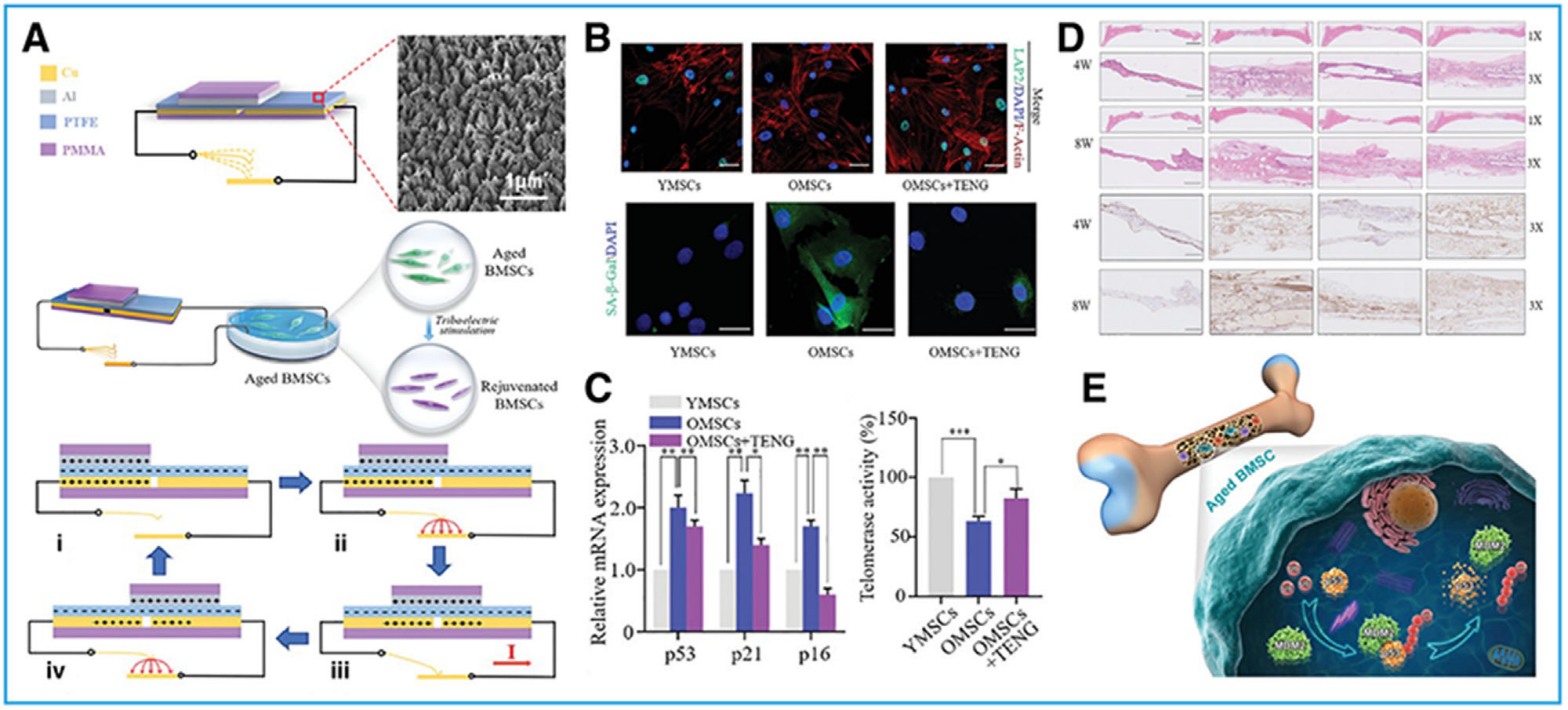
Effects of ES generated by triboelectric nanogenerators (TENGs) on cell rejuvenation. (A) Schematic illustration and working principle of a pulsed triboelectric nanogenerator (P‐TENG). (B) Fluorescence staining of BMSCs and their expression products. (C) Expression levels of p53, p21, and p16. (D) Osteogenic regeneration of senescent BMSCs shown by H&E staining and ALP IHC. (E) Schematic mechanisms of electrical stimulation in aged BMSCs via MDM2‐P53. Reproduced with permission.^[^ ^73^ ^]^ Copyright 2021, Wiley‐VCH GmbH.

Cell senescence, a mechanism of cell‐cycle arrest that cannot be reversed, is related to cell phenotypic alterations, persistent DNA damage, tumor suppressor activation telomere attrition, and so on. It is not only a self‐protection mechanism for preventing cancer, but also may be associated with some age‐related diseases.^[^ ^68^, ^69^ ^]^ Therefore, selectively removing harmful senescent cell populations, shutting down their secretory machinery, or restoring the vitality of senescent cells and promoting cell rejuvenation by regulating the interactions between metabolic processes and epigenetic modification can delay age‐related degenerative diseases, which is beneficial for human health and life extension.^[^ ^70^, ^71^, ^72^ ^]^ Li et al.^[^ ^73^ ^]^ created a pulsed triboelectric nanogenerator (P‐TENG) (Figure 10A), which could generate ES to rejuvenate senescent BMSCs. The two electrodes of the P‐TENGs were made of Cu foil, and a square of Al foil as the triboelectric layers on the surface of a polymethyl methacrylate (PMMA) sheet and a polytetrafluoroethylene (PTFE) film attached to the copper foil. Under the ES action of these P‐TENGs at 30 μA and 1.5 Hz, BMSCs underwent a phenotypic transformation with changes in senescence biomarkers expression; associated proteins; and enzymes related to osteogenesis, chondrogenesis, and proliferation; transcription factors essential for the pluripotency maintenance; and so on. The BMSCs proliferation was significantly enhanced, with increased stemness and higher activities of osteogenesis and chondrogenesis (Figure 10B–E). These results provide more possibilities for the application of P‐TENGs in the treatment of some orthopedic diseases, and if they can be further developed, P‐TENGs may become an efficient tool for repairing and regenerating more tissue.^[^ ^73^ ^]^

## 4 CHALLENGES AND FUTURE PROSPECT FOR CELL STIMULATION WITH TENGS

TENGs are efficient energy conversion devices that can utilize mechanical energy taken from the surrounding environment and transform it into electrical energy. For example, they are able to obtain mechanical energy from the movement of organisms, convert it into ES, and then act on the organisms themselves to produce bioelectric effects and change cellular activities.^[^ ^15^, ^16^ ^]^ Owing to their advantages of easy manufacture, low cost, lightweight, and high efficiency, TENGs have various applications in the self‐powered treatment of respiratory, cardiovascular, neurological, and musculoskeletal diseases; electroporation‐based drug delivery; real‐time human health monitoring; minimally invasive accurate diagnosis; etc.^[^ ^19^, ^20^, ^21^, ^22^ ^]^

From this article, we summarized the principles and characteristics of TENGs, reviewed their research progress in the field of cell stimulation, focused on their effects on the processes of cell function regulation (alignment, migration, secretion, and membrane permeability change) and cell fate determination (proliferation, differentiation, reprogramming, and rejuvenation), and stressed on the application potential of TENGs in various processes. As TENGs are a type of emerging high‐tech equipment, most studies are still in the theoretical and laboratory stages, and there are still certain milestones to achieve before their formal clinical application. TENGs are widely used in neurology, regeneration therapy, rehabilitation, pharmacy technology, and other fields and still have certain opportunities and challenges.

i Producing stable and accurate stimulation signals.

Different intensities of ES have different effects on cells. There is often an optimum ES intensity for cellular function regulation and fate determination. As mentioned earlier, if the stimulation is not limited, it will also cause damage to the cells themselves and surrounding tissues.^[^ ^54^ ^]^ Therefore, finding the ES that has the best effect on cell activity and the least harm to the body often requires researchers’ continuous exploration and repeated attempts.

ii Selecting the appropriate mechanical energy source.

At present, the commonly used methods of energy harvesting are mainly the environmental energy (such as wind energy, acoustic energy), the mechanical movement of organisms (such as the bending of the spine, the motion of limbs) and the physiological movement of organs (such as respiratory movement and heart beating).^[^ ^18^ ^]^ For example, in order to promote wound healing, TENGs designed by long et al. used respiratory movement, which should be the main source of mechanical energy for trauma patients during bed rest.^[^ ^5^ ^]^ In addition, Han et al. used TENGs to make a cardiac pacemaker, which was inserted into the pericardium to obtain energy from the heartbeat and had higher requirements for miniaturization and histocompatibility because of its implantability.^[^ ^74^ ^]^ Thus, the use of TENGs should be fully considered in the selection of mechanical energy source.

iii Optimizing and improving reliability and facilitating mass production.

Since TENGs will be used as wearable or implantable devices in practical applications, the requirements of their reliability, biocompatibility, and long‐term biosafety must be very strict. TENGs should have the characteristics of small size, strong tolerance to complex environments, and long‐term operation.^[^ ^16^ ^]^ In terms of material optimization, high triboelectric performance, long‐term existence in vivo and environment‐friendly materials should be selected. The cytotoxicity of the incorporated materials should be studied and tested regularly.^[^ ^75^ ^]^ Several teams have already developed biodegradable materials for TENGs, which are very helpful for improving biocompatibility.^[^ ^76^ ^]^

iv Human application and clinical translation.

At present, research on TENGs in disease monitoring, diagnosis, and treatment mostly remains in the laboratory stage, mostly at the level of tests in vitro or in rat models. For example, in vitro tests lack many influencing factors of in vivo tests, such as a complex extracellular environment and interactions with other cells and tissues.^[^ ^77^ ^]^ Moreover, for wound healing, the different healing modes and degrees of wound contraction among different species will reduce the universality of the results.^[^ ^78^ ^]^ More in vivo tests and clinical studies are needed in the future to realize the translation of TENG application from cells to complete organisms, from rats to primates, and from the laboratory to the clinic, which ultimately will be a long process.

v Intelligence and informatization of TENGs.

With the progress of the world, everything is becoming intelligent, and so are wearable and implantable devices. In clinical application, these devices should be able to perform the function of the real‐time monitoring of the various data of the human body so that doctors and patients will have immediate and real‐time access to their health‐care information. When the new generation TENGs are used for disease treatment, they should also be capable of the adjustment of ES parameters intelligently and in real time according to this information to achieve the purpose of individualized treatment. In addition, coupled with the emergence of the 5G network and IoT, the TENG application will allow clinicians to more conveniently and quickly adjust the treatment plans of patients.^[^ ^79^ ^]^

The regulation of various cellular processes by TENGs has been described in detail in this review. At present, many research teams are exploring the therapeutic role of cell stimulation in several diseases,^[^ ^80^, ^81^ ^]^ such as nerve regeneration,^[^ ^66^, ^67^ ^]^ osteogenesis,^[^ ^10^, ^62^, ^82^ ^]^ wound healing,^[^ ^5^, ^50^, ^59^, ^83^ ^]^ hair regeneration,^[^ ^49^ ^]^ myocardium and skeletal muscle function rehabilitation,^[^ ^84^, ^85^ ^]^ etc. Even some therapeutic effects of ES have been recognized by several authoritative international clinical guidelines,^[^ ^86^, ^87^ ^]^ which has also led to the mushrooming development of tissue engineering and regenerative medicine.^[^ ^88^, ^89^, ^90^ ^]^ In the future, there will be more research on the treatment of TENGs‐induced cellular ES, and it is believed that one day the devices will be able to be put into clinical application.

In conclusion, the electrical stimulation generated by TENGs has great development prospects for influencing cell function and fate. TENGs have been researched steadily for nearly 10 years. From this review, we summarized the principles and characteristics of TENGs, analyzed their research progress in the field of cell stimulation, and provided a general direction for the development of TENGs in this field. As mentioned in this article, TENGs facilitate multiple medical applications and development opportunities at the level of prevention, therapy, and rehabilitation. The development direction of each field has various possibilities. Thanks to their advantages, TENGs may come to revolutionize the medical device industry and have a boundless future.

## AUTHOR CONTRIBUTIONS

Xingyu Zhou, Gaocai Li, Di Wu, and Huaizhen Liang wrote this manuscript and contributed equally to this manuscript. Weifeng Zhang, Lingli Zeng, Qianqian Zhu, and Puxiang Lai helped check this manuscript. Zhen Wen and Yue Pan supervised the preparation of this manuscript, and Cao Yang and Yue Pan conceived and directed the research.

## CONFLICT OF INTEREST STATEMENT

The authors declare no conflict of interest.
